# Supplementary material for: Gut hormone signaling drives sex differences in metabolism and behavior
Source: Mol Metab. 2025 Dec 19;103:102312. doi: 10.1016/j.molmet.2025.102312 (PMC12808592; doi:10.1016/j.molmet.2025.102312)
Supplement: Multimedia component 6 [file mmc6.pdf]

Supplementary Figures

Gut hormone signaling drives sex differences in metabolism and behavior

Olga Kubrak, Alina Malita, Nadja Ahrentløv, Stanislav Nagy, Michael J. Texada, Kim Rewitz

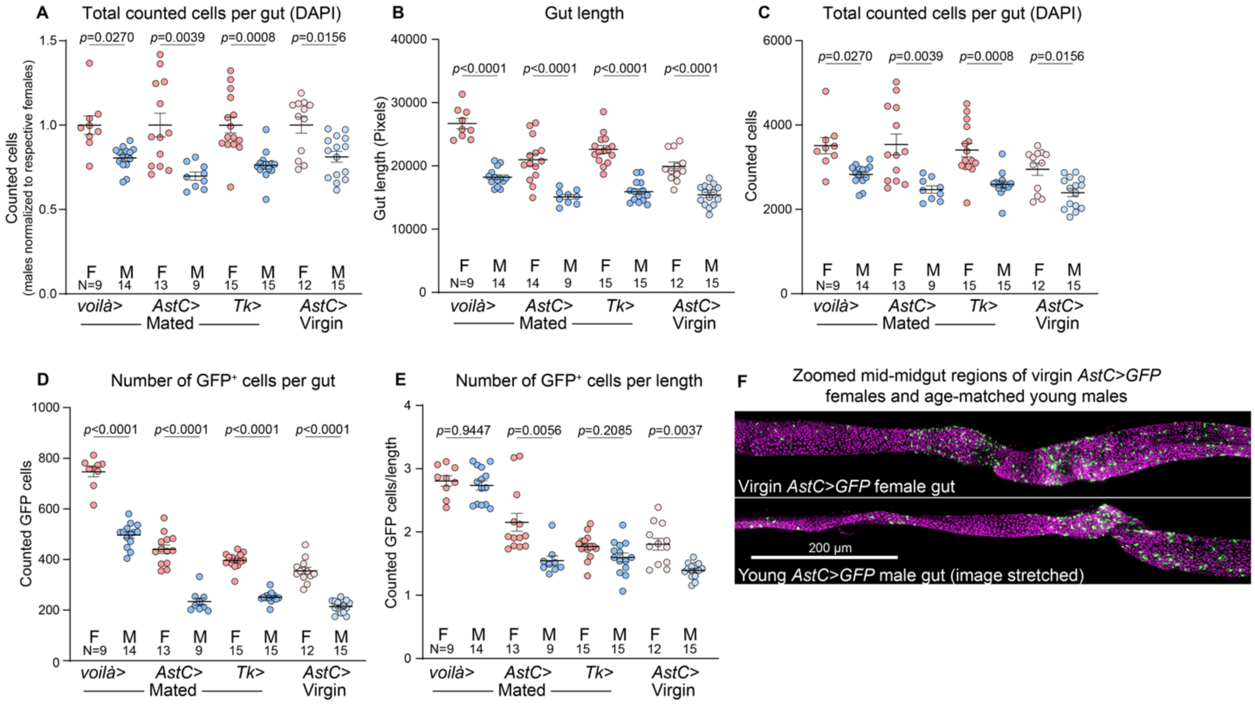

Supplementary Figure 1. Gut length, total cell number, and EEC counts. (A) The total number of cells of all kinds per midgut (detected with DAPI staining). Values for each sex and genotype are normalized to the female average. (B) Absolute midgut length (measured in pixels in the confocal image) in males ("M") and females ("F") expressing GFP in defined enteroendocrine-cell (EEC) populations using *voilà-GAL4*, *AstC::T2A::GAL4* (*AstC*>), or *Tk::T2A::GAL4* (*Tk*>) drivers, or *AstC*> in virgins. (C) Total number of cells of all kinds per midgut (DAPI-stained nuclei), not normalized. (D) Number of GFP-positive EECs per gut. (E) Number of GFP-positive EECs per unit length, shown without normalization to the female reference values. Data are presented as mean ± s.e.m. with individual data points. Statistical comparisons between sexes are indicated. Statistics: A-D: Welch's ANOVA with Dunnett's T3 multiple comparisons. All experiments used mated females.

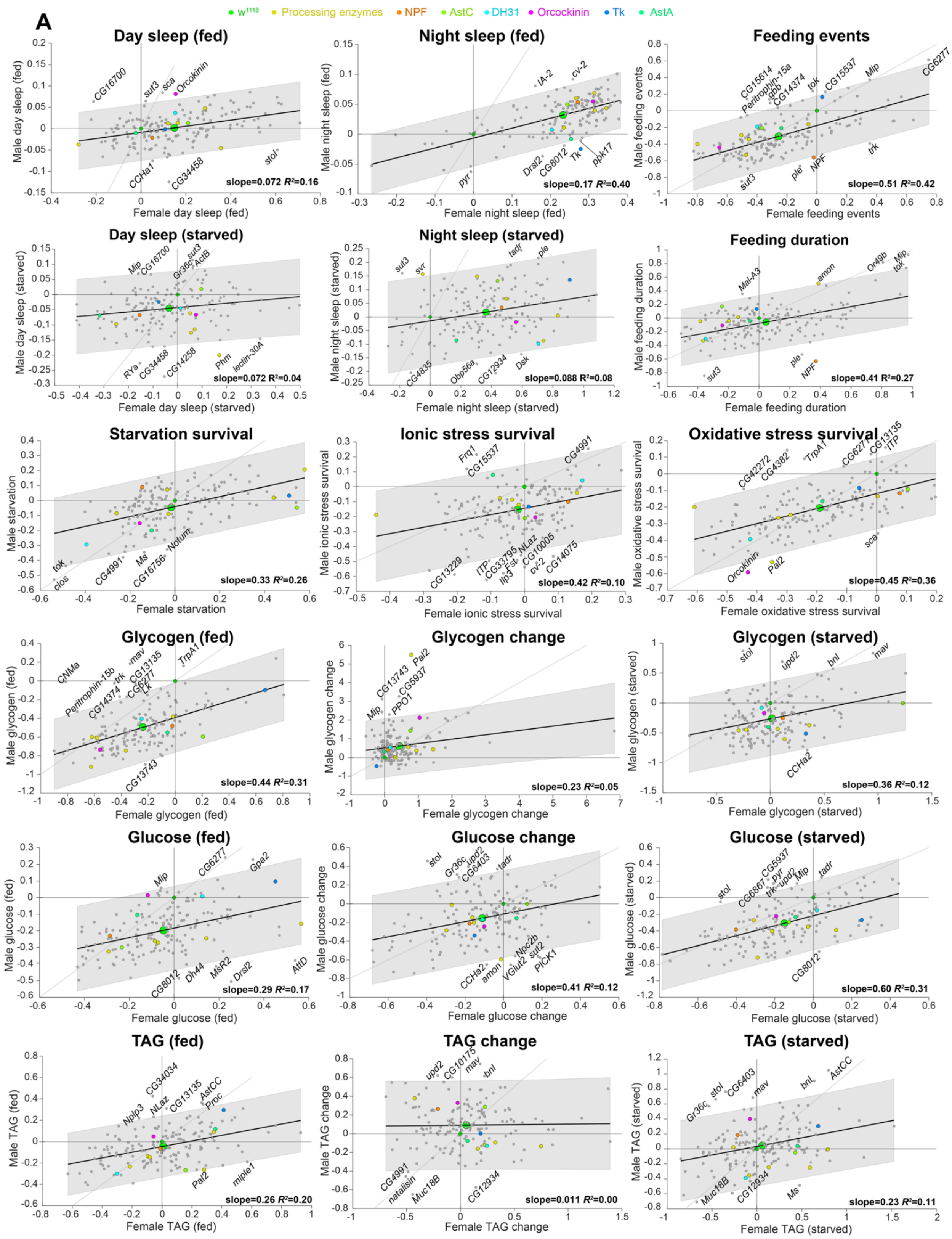

Supplementary Figure 2. Comparative analysis of male and female responses across all physiological and behavioral assays. (A) Plots of each knockdown genotype's effects in females (X axis) versus in males (Y axis). Each

point represents an individual gene knockdown, with a fitted trend line and 95% confidence intervals shown in gray. The control genotype falls at position (0, 0) by definition, and the mean of all genotypes is indicated as a larger green dot. Points along the diagonal represent knockdowns with similar effects in both sexes, and distance from the diagonal indicates the degree of sex bias in effect size. Points in opposite quadrants, where the effect is positive in one sex but negative in the other, represent strong discordance, in which a given knockdown elicits opposing phenotypes in males and females. In the metabolite-loss charts, positive values indicate greater depletion during starvation than in the control, 0.0 indicates losses equal to those of the control, and negative values indicate smaller losses than in the control; a score of –1.0 indicates no loss during starvation (100% less than in the control). All experiments used mated females.

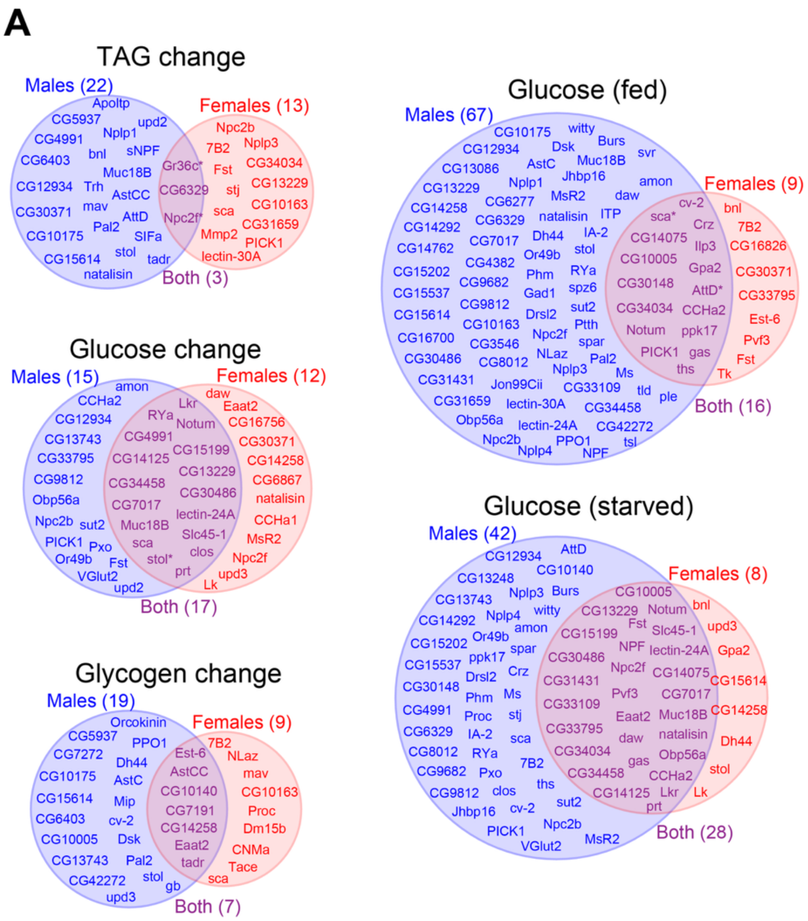

Supplementary Figure 3. Sex-specific gut hormone knockdown phenotypes in additional metabolic assays. Hits were defined as EEC gene knockdowns with phenotypic values outside ±1.5 standard deviations (SD; z-score ±1.5) from the control distribution. This definition was applied consistently across all panels. (A) Venn diagrams show the distribution of hits into male-specific (blue, left), female-specific (red, right), and shared (purple, overlap) categories. Panels display hits for TAG loss during starvation, glucose levels in fed and starved animals, and glucose and glycogen loss during starvation. All experiments used mated females.

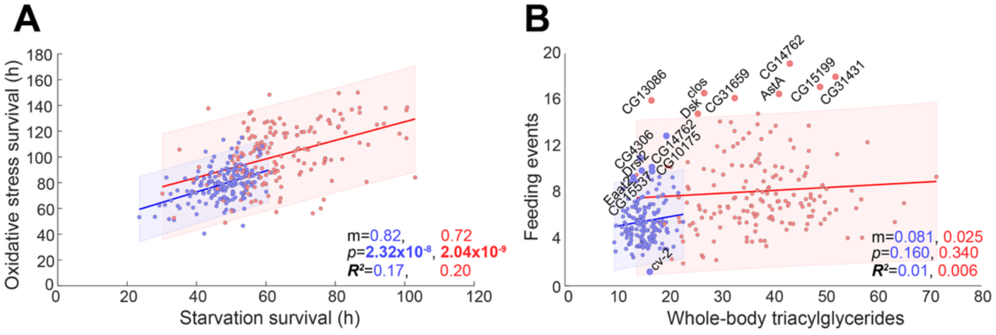

Supplementary Figure 4. Additional trait correlations involving survival and feeding. (A) Starvation-survival duration plotted against oxidative-stress survival time across EEC-specific knockdown lines in females (red) and males (blue). (B) Whole-body triacylglyceride (TAG) levels plotted against number of feeding events. Each dot represents an individual knockdown line. Trend lines are shown with shaded 95% confidence intervals. Knockdowns falling outside this region are labeled. Reported values indicate each fit's slope (m), *p*-value for "no correlation," and coefficient of determination (R^2) for males (blue, left) and females (red, right). All experiments used mated females.

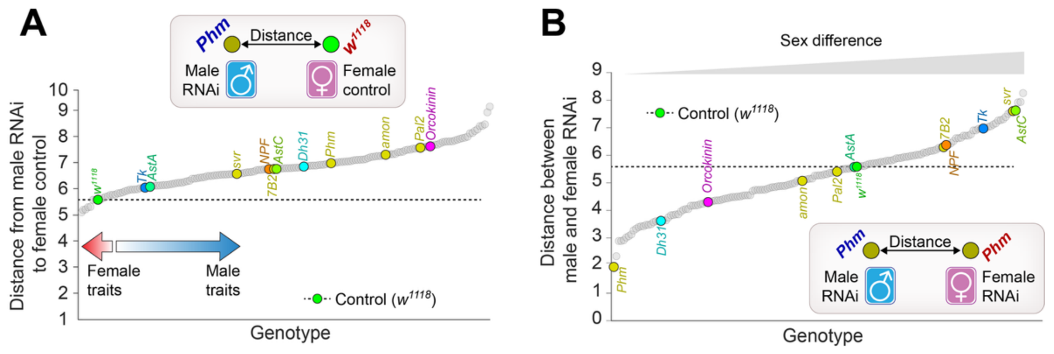

Supplementary Figure 5. Sex differences in phenotypic space across EEC knockdowns. (A) Euclidean distance in the PCA1 x PCA2 plane from each male knockdown to the female control. The schematic inset illustrates how the distance was calculated between male RNAi samples and the female control. The control (*voilà*> x w^{1118}) is shown in green. (B) Euclidean distance in the PCA1 x PCA2 plane between male and female RNAi animals of the same genotype. Genotypes are ordered along the x-axis, with the control (*voilà*> x w^{1118}) indicated in green. Selected knockdowns are labeled. The schematic inset illustrates the distance calculation between male and female RNAi pairs. Statistics: Phenotypic values were converted into Z-scores, and PCA was performed with centering and the singular value decomposition method. All experiments used mated females.

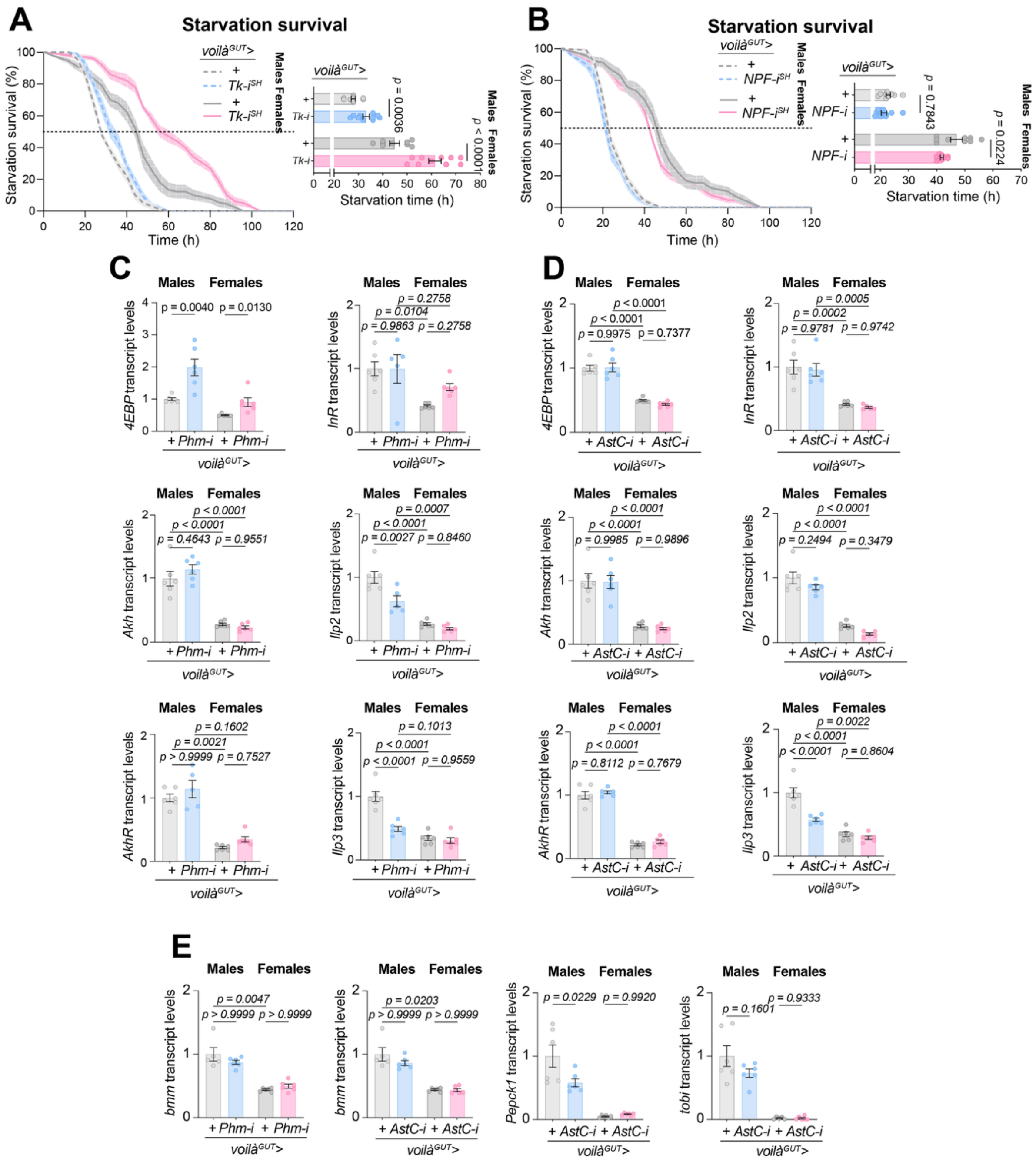

Supplementary Figure 6. Additional effects of EEC-derived peptide-hormone knockdown on stress survival and metabolic gene expression. EEC specificity was achieved with *voilà*^{GUT}> (*voilà-GAL4* combined with the pan-neuronal repressor *R57C10-GAL80* restricting RNAi to gut EECs, and with *Tub-GAL80*^{TS} to temporally restrict knockdown to the adult stage). (A, B) Survival under starvation upon EEC-specific knockdown of *Tk* (A) or *NPF* (B). (C, D) Expression of insulin- and AKH-pathway components (*Ilp2*, *Ilp3*, *Akh*, *AkhR*, *InR*, *4EBP*) in males and females upon EEC-specific knockdown of *Phm* (C) or *AstC* (D). (E) Expression of metabolic regulators (*bmm*, *Pepck*, *tobi*) in males and females upon EEC-specific knockdown of *Phm* or *AstC*. Bar graphs show mean ± s.e.m. Statistical tests: survival assays (A, B) were analyzed by one-way ANOVA with Tukey's post-hoc tests, and and gene-expression assays (C-E) were tested using one-way ANOVA with Tukey's post hoc tests or unpaired two-sided *t*-test (pairwise comparisons). All experiments used mated females.
